# Now That We Are Disaggregating Race and Ethnicity Data, We Need to Start Understanding What They Mean

**DOI:** 10.1001/jamanetworkopen.2024.3674

**Published:** 2024-05-01

**Authors:** Joshua J. Quint, Joseph Keawe’aimoku Kaholokula

**Affiliations:** Papa Ola Lokahi, Honolulu, Hawai‘i; Department of Native Hawaiian Health, John A. Burns School of Medicine, University of Hawai‘i at Manoa, Honolulu

The study by Santi and Verhoef^1^ examined racial and ethnic disparities for in-hospital COVID-19 mortality in Hawai‘i using disaggregated data. The study by Santi and Verhoef^1^ highlights the importance of disaggregating Asian, Native Hawaiian, and Pacific Islander individuals in clinical research. It also provides an opportunity to discuss some of the nuances and complexities involved with data disaggregation.^1^

## Data Disaggregation Standards

Representing some of the fastest growing race and ethnicity groups and with a population of more than 25 million in the United States, there is an increasing need for disaggregated Asian, Native Hawaiian, and Pacific Islander data. Since 1997, federal data standards have separated Asian individuals and Native Hawaiian and Pacific Islander individuals into distinct categories.^2^ The US Department of Health and Human Services recommended the collection of detailed data for Asian and Native Hawaiian and Pacific Islander populations in 2011, adopting the detailed list used in the 2010 Census. Yet, many studies continue to combine Asian, Native Hawaiian, and Pacific Islander data into a single group, and very few studies use more detailed categories. Aggregating diverse race and ethnicity groups can conceal underlying disparities and is a barrier to health equity.^3^ When aggregate statistics are reported, heterogeneity in the experiences of smaller groups become invisible, which can inhibit the ability of these communities to advocate for resources.

The value of disaggregated data is readily apparent in Hawai‘i, where more than two-thirds of the population identifies as Asian, Native Hawaiian, or Pacific Islander. The distinct cultural, historical, and socioeconomic backgrounds of these populations contribute to varying life expectancies and other health disparities.^4^ Specificity (ie, level of detail) and context (eg, social determinants of health) are important factors to consider when disaggregating data to identify and address health inequities. Social epidemiology and causal inference methods are valuable tools when going beyond mere descriptions of health disparities to identify root causes and understand the social determinants of health.

On March 28, 2024, the US Office of Management and Budget published a historic revision to the 1997 Statistical Policy Directive, aimed at improving federal race and ethnicity statistics and ensuring that data more accurately reflect the racial and ethnic diversity of the US population. The standard now includes a requirement to collect more detailed categories and for tabulation procedures to result in the production of as much information as possible. With regards to persons who select multiple race or ethnicity categories, the standard describes the nonmutually exclusive alone or in combination approach for tabulation and reporting. Since we advocate here for the use of more detailed categories and the alone or in combination approach, this commentary may serve as a timely and useful resource for agencies and researchers seeking to implement the new standards.

## Collecting Granular Race and Ethnicity Data

The study by Santi and Verhoef^1^ goes well beyond the minimum standard by further disaggregating among Asian, Native Hawaiian, and Pacific Islander groups (eg, Chinese, Filipino, Japanese, Native Hawaiian, and Samoan) and by including multiracial persons in each race and ethnicity category. The hospital facilitated this level of detail by providing 20 options for patient race and ethnicity at enrollment and presentation for clinical care. The collection of such granular race and ethnicity data facilitates more nuanced approaches to health disparities and the use of race and ethnicity as proxies for some of the unmeasured drivers of health disparities.

## Analyzing Multiracial Data

The classification of persons with more than 1 racial or ethnic identity represents a challenge in statistical analysis. Santi and Verhoef^1^ refer to the single–race and ethnicity statistic of 11% for Native Hawaiian or Pacific Islander individuals when describing the demographic characteristics of the state population, when 27% of the state population identifies as Native Hawaiian or Pacific Islander.^4^ By comparing the race and ethnicity subgroup counts with the total study population number, we can infer that the race and ethnicity groupings were not mutually exclusive and that some patients were represented more than once in some analyses.

Using nonmutually exclusive race and ethnicity categories is appropriate and preferrable in many instances. For example, the Census Bureau provides data for both alone and alone or in combination for detailed race and ethnicity categories, which can also serve as population denominators for disparity estimates. This inclusive approach to race and ethnicity is also consistent with the federal legislation defining Native Hawaiian individuals as anyone with ancestral origins in the Hawaiian Islands prior to 1778.^4^ Attempts to create mutually exclusive groupings that include a multiracial category should be weighed against the resulting loss of information, and in many cases, using an alone or in combination category may be preferrable to the single–race and ethnicity approach. Researchers can avoid potential misinterpretations by clearly explaining how multiracial persons are classified in the analysis.

## Hospital-Based vs Population-Based Mortality Rates

While hospital-based mortality rates provide valuable insights into inpatient outcomes, they may not fully capture broader population-level trends in COVID-19 mortality. For instance, Pacific Islander populations consistently experienced the highest age-adjusted mortality rates during the pandemic in Hawai‘i. However, these disparities were not fully reflected in the study by Santi and Verhoef,^1^ in which Pacific Islander populations surprisingly experienced lower mortality rates across several strata of analysis.^5^ Conversely, findings by Santi and Verhoef^1^ regarding Filipino, Native Hawaiian, and Japanese populations largely aligned with external data, albeit with notable exceptions observed among Native Hawaiian populations during the Delta wave. According to state vital statistic data, Native Hawaiian populations had age-adjusted mortality rates lower than those of the general population in 2020 (14 deaths per 100 000 population), but rates subsequently increased in 2021 during the Delta wave (61 deaths per 100 000 population). The population-based COVID-19 mortality rates among Native Hawaiian populations in 2021 were therefore higher than those among the overall population of Hawai‘i (35 deaths per 100 000 population) and that of Filipino populations (54 deaths per 100 000 population), trailing only Pacific Islander populations (284 deaths per 100 000 population), which was not reflected in the population from the study by Santi and Verhoef.^1,5^ The apparent inconsistencies between the findings by Santi and Verhoef^1^ and population-based Native Hawaiian and Pacific Islander mortality data are not surprising when considering the unique setting of the study, the restriction to a specific component of mortality risk, and the impacts of statistical adjustment.

## Nonrepresentativeness and Health Care Factors

There are many complex factors influencing COVID-19 mortality that extend beyond the hospital setting, making hospital-based studies an incomplete representation of overall mortality trends. This study by Santi and Verhoef,^1^ focused on patients seeking care for complications within a single health care facility, inherently captures only a subset of mortality risk over a limited timeframe. Moreover, statistical adjustments can inadvertently produce associations that have less relevance for the actual population if they cannot modify for other factors, like body mass index, age, sex, type of insurance, comorbidities, and neighborhood conditions. These concerns can be addressed by adhering to established epidemiologic guidelines, such as reporting unadjusted outcome statistics for cohort studies.^6^ Additionally, specifying the reference group and the hypothesized causal model can further increase the interpretability of adjusted race and ethnicity statistics while also mitigating concerns surrounding nonrepresentativeness.

In-hospital mortality can be an indicator of the quality of medical care provided, especially if patients have similar age and health status at the time of admission. Race and ethnicity disparities for in-hospital mortality that remain after adjustment for other potential risk factors may then reflect individual and process-level quality of care factors that are influenced by a patient’s race and ethnicity.^7^ For example, race and ethnicity discordance between patients and clinicians may interact with implicit bias among health care workers to create disparities in hospital-based outcomes. Santi and Verhoef^1^ point to the absence of an association between insurance type and mortality as encouraging evidence of equitable care delivery for people with different insurance types but miss the opportunity to discuss how the presence of associations might point to the provision of inequitable hospital care on the basis of race and ethnicity.

## Conclusions

Disaggregation is a crucial step in the journey toward collecting and reporting data that better reflect the social and cultural contexts associated with health disparities. Santi and Verhoef^1^ contribute to this discourse by using detailed and inclusive race and ethnicity categories in their hospital-based study of COVID-19 mortality. Once data have been disaggregated, interdisciplinary collaboration across relevant fields, including social sciences and epidemiology, can provide helpful tools to address the complexities and nuances of detailed race and ethnicity data. With disaggregated data properly placed into social contexts, we will be better equipped to develop health care policies and resource allocation strategies aimed at addressing health inequities and promoting equitable access to care.
